# Socioeconomic Factors in Patients with Ulnar Nerve Compression at the Elbow: A National Registry-Based Study

**DOI:** 10.1155/2020/5928649

**Published:** 2020-12-18

**Authors:** Malin Zimmerman, Erika Nyman, Katarina Steen Carlsson, Lars B. Dahlin

**Affiliations:** ^1^Department of Translational Medicine-Hand Surgery, Lund University, Skåne University Hospital, Jan Waldenströms gata 5, S-205 02 Malmö, Sweden; ^2^Department of Hand Surgery, Skåne University Hospital, Jan Waldenströms gata 5, S-205 02 Malmö, Sweden; ^3^Department of Biomedical and Clinical Sciences, Linköping University, 581 83 Linköping, Sweden; ^4^Department of Hand Surgery, Plastic Surgery and Burns, Linköping University Hospital, 581 85 Linköping, Sweden; ^5^Department of Clinical Sciences, Malmö, Lund University, 223 81 Lund, Sweden

## Abstract

**Aims:**

To investigate demographics and socioeconomic status in patients with ulnar nerve compression and the influence of socioeconomic factors on patient-reported outcome measurements (PROM) as evaluated by QuickDASH (short version of Disabilities of Arm, Shoulder and Hand) after surgery for ulnar nerve compression at the elbow.

**Methods:**

Patients operated for primary ulnar nerve compression from 2010 to 2016 were identified in the National Quality Registry for Hand Surgery Procedures (HAKIR). Patients filled out questionnaires before and at three and 12 months after surgery. A total of 1346 surgically treated cases were included. Data from HAKIR were linked to data from Statistics Sweden (SCB) on socioeconomic status (i.e., education level, earnings, social assistance, immigrant status, sick leave, unemployment, and marital status).

**Results:**

Patients surgically treated for ulnar nerve compression at the elbow differed from the general population with lower levels of education, higher social assistance dependence, a high proportion of unemployment, and lower earnings. However, the results were not clear concerning the influence of socioeconomic factors on the outcome of surgery, except for long-term sick leave.

**Conclusion:**

Patients surgically treated for ulnar nerve compression at the elbow are socioeconomically deprived, but only a history of long-term sick leave influences the outcome of surgery. This information is crucial in the diagnosis and treatment of these patients.

## 1. Introduction

Ulnar nerve compression is the second most common nerve entrapment in the upper extremity [1], with a reported incidence of 25-30 per 100,000 person-years [2, 3]. Known risk factors include smoking [4] and occupational factors [5]. Sex distribution varies between studies [3, 6], and there is often considerable comorbidity [5–7]. There is no clear consensus nor evidence regarding when ulnar nerve compression at the elbow is best treated conservatively and when surgery is indicated [1], a procedure that may induce complications, including neuropathic pain [6]. Often, milder symptoms are treated conservatively, and more severe symptoms or motor impairment are treated surgically.

When using patient-reported outcome measurements (PROM), such as the QuickDASH (short form of Disabilities of the Arm, Shoulder and Hand), outcomes do not seem as favorable as for other surgeries, such as carpal tunnel release [1], but the reason for this is unknown.

Socioeconomic factors are known to affect an individual's perception of health as well as outcome after surgery and other illnesses. It has been extensively studied in total joint arthroplasty and orthopedic trauma surgery, where socioeconomic factors affect postoperative results and patient health [8]. In ulnar nerve compression at the elbow, a low education level is a risk factor development [5], but whether socioeconomic factors influence patients' preoperative symptoms or affect outcome after surgical treatment for ulnar nerve compression is unknown. The aim of this study was to investigate demographics and socioeconomic status in a population surgically treated for ulnar nerve compression and whether socioeconomic factors affect the outcome of surgery.

## 2. Methods

### 2.1. Study Population

We identified patients surgically treated for ulnar nerve compression (using ICD-10 code G562 and primary surgical codes (KKÅ97) ACC53, ACC43, or NCK19) in the National Quality Registry for Hand Surgery procedures in Sweden (HAKIR) during the period from 2010 to 2016. Patients above 16 years of age that are able and willing to provide informed consent are included in the registry. In the present study, only patients ≥ 18 years of age and primary surgeries were included. Patients were asked to fill out the Swedish version of the QuickDASH questionnaire [9] before surgery, and at three and 12 months after surgery, either online or by traditional mail, with a reminder by text if not answered within 48 hours. The QuickDASH contains 11 questions, where the patient is asked to score disability in the affected arm. A total score ranging from 0 to 100, where 100 represents the worst possible disability, is calculated. Data from HAKIR was combined with socioeconomic data from Statistics Sweden (SCB) using personal identification numbers, and with the Swedish National Diabetes Register (NDR) for diabetes status.

### 2.2. Socioeconomic Factors

Data on earnings were available from 1990 to 2016. Mean earnings during these years was calculated as mean earnings for the years that the patient was between 30 and 65 years of age to get an estimate of the earnings during working years (65 years was in 2010-2016 the common retirement age in Sweden). A binned variable was created with 25% of the patients in each group. Earnings were indexed to December 2016 using the consumer price index.

Sick leave was calculated as net days, and only sick days exceeding 14 calendar days are included due to the way reimbursement is organized in Sweden, where the employer pays for the first two weeks of sick leave. Only citizens that have been employed during the last six months are entitled to paid sick leave. A mean number of sick days were calculated as mean sick days per year of employment and were above 20 years of age during the period that data was available (1994-2016).

Social assistance data was available from 1990 to 2016. Social assistance was individualized from family. We calculated a new variable based on whether the patient had received social assistance once during these years, several times, or never.

Unemployment data was available from 1992 to 2016 and calculated as mean days per year. We also calculated one variable corresponding to whether the patient was employed during the year of surgery or not.

Marital status data was available from 1990 to 2016 and was recorded from the year of surgery. These data included if the patient was married, registered partner, divorced or divorced partner, or widowed.

Educational level was based on the education level that the patient had during the year of surgery. Levels were based on the International Standard Classification of Education (ISCED) [10], as follows: primary: ISCED 0, 1, and 2 (≤9 years of education, compulsory school); upper secondary: ISCED 3 (9-12 years of education); and tertiary: ISCED 4, 5, and 6 (>12 years of education).

### 2.3. Statistical Analyses

A binned variable was created for age, based on equal percentiles (25% of the population in each age group). Nonparametric data is presented as median (interquartile range, IQR). Nominal data is presented as number (%) and was compared using the chi-squared test. The Mann-Whitney *U*-test was used to compare data when there were two groups, and the Kruskal-Wallis test was used for comparisons between more than two groups. Subsequent pairwise comparisons were adjusted by the Bonferroni corrections for multiple tests. To investigate the effect of socioeconomic factors on QuickDASH scores, we used multivariate linear regression analysis. Each variable was analyzed separately in model 1. In model 2, we analyzed all variables separately but adjusted for age at surgery, sex, and diabetes at surgery. In model 3, all variables were included and adjusted for age, sex, and diabetes at surgery. In the reduced model 1 [11], we included all variables with a *p* value < 0.3 in model 3. In the reduced model 2, we included all variables with a *p* value < 0.1 in reduced model 1.

We considered each treated arm a separate statistical entity. We considered a *p* value of <0.05 statistically significant. All calculations were performed using IBM SPSS Statistics version 24 or 25 (SPSS Inc., Chicago, IL).

### 2.4. Ethics

The Regional Ethical Review Board in Lund, Sweden, approved this study (2016/931, 2018/57 and 2018/72).

## 3. Results

During the study period, we identified 1278 individuals (1346 arms), who met the inclusion criteria and were surgically treated for ulnar nerve compression at the elbow. The study population has been described previously [12]. The majority of patients were treated with simple decompression (1167/1346; 86%), 150/1346 (11%) were treated with transposition of the ulnar nerve, and 32/1346 (2%) were treated with medial epicondylectomy.

### 3.1. Response Rates

Preoperatively, 452/1354 (33%) cases responded to the QuickDASH; at three months postoperative, 311/1285 (24%) responded; and at 12 months postoperative, the response rate was 294/1089 (27%). Age, sex, and diabetes prevalence did not differ between responders and nonresponders preoperatively, and at 12 months postoperative, respondents were older and more often female (data already published, [12]).

### 3.2. Age

Socioeconomic factors in different age categories are presented in Table 1. In the youngest group (18-42 years), there were more men than in the other groups. The lowest proportion of immigrants was found in the oldest age group (>63 years). The oldest group also had the lowest number of sick days, days as unemployed, and the least dependence on social assistance (Table 1).

### 3.3. Socioeconomic Factors

During the year of surgery, 435/1346 (32%) cases were not married, 571/1346 (42%) were married, 289/1346 (22%) were divorced, and 46/1346 (3%) were widowed. In the linear regression analysis, being divorced reduced postoperative QuickDASH scores at 12 months (Table 2). This effect remained in the reduced models (Tables 3 and 4). Data was missing in 5 (0.4%) cases.

**Figure 1:**
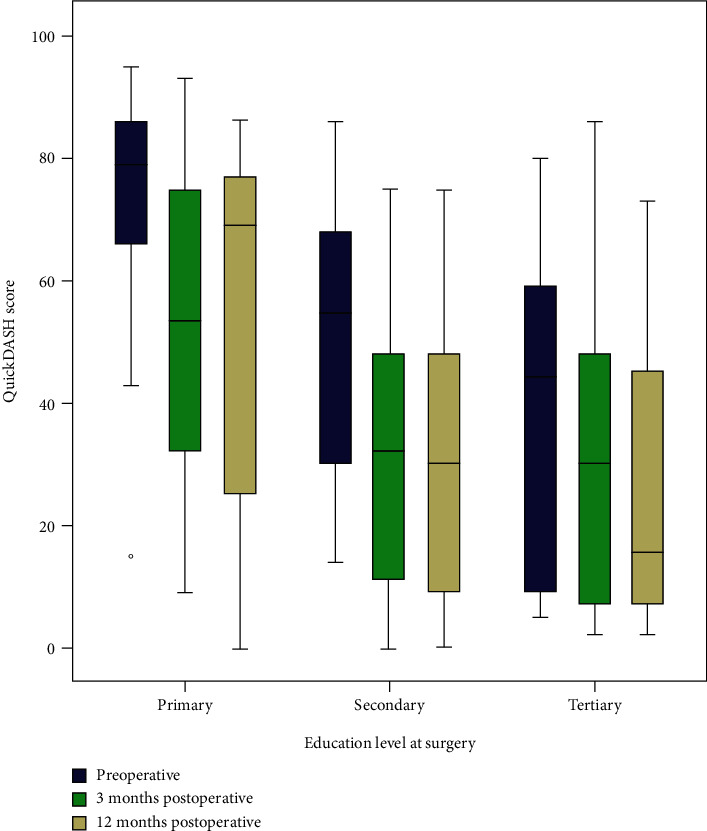
QuickDASH scores before and at three and 12 months postoperative in surgically treated cases of ulnar nerve compression at the elbow. Comparison of groups based on education level.

In the study population, 330/1346 (25%) had >12 years of education (Table 1). The group with the lowest level of education scored highest on all occasions in the QuickDASH (Figure 1). A high education level reduced the postoperative QuickDASH score at 12 months when adjusting for age, sex, and diabetes. In model 3, adjusting for all other socioeconomic factors as well, education level was no longer statistically significant (Table 2). Data on education level was missing in 10 (0.8%) cases.

**Figure 2:**
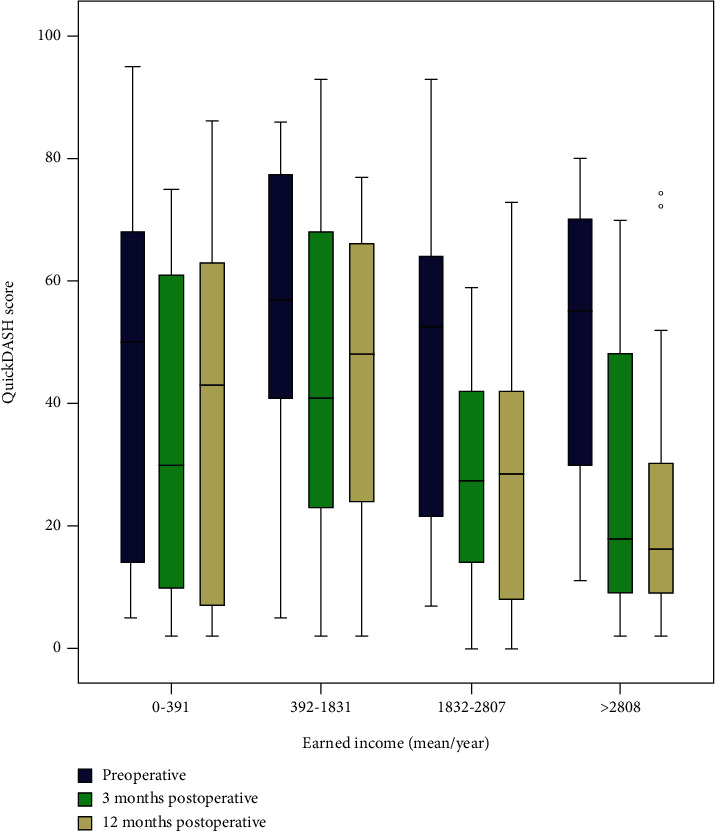
QuickDASH scores before and at three and 12 months postoperative in surgically treated cases of ulnar nerve compression at the elbow. Comparison of groups based on income level. Earned income presented as 1000 Swedish kronor (SEK).

Postoperative QuickDASH scores were lowest in the highest-earning group (Figure 2). Higher earnings reduced postoperative QuickDASH scores at 12 months in the linear regression analysis when adjusting for age, sex, and diabetes. However, when also adjusting for other socioeconomic factors, earnings were no longer a statistically significant determinant of postoperative QuickDASH scores (Table 2).

In the study population, 224/1346 (17%) were born outside of Sweden (Table 1). Migrant status did not affect postoperative QuickDASH scores (Table 2).

During the year of surgery, 530/1346 (40%) had work that was classified as manual. Manual work did not affect postoperative QuickDASH scores (Table 2).

Low sick leave reduced the postoperative QuickDASH scores at 12 months in linear regression models 1 and 2 but did not remain statistically significant when adjusting for other socioeconomic factors in model 3 (Table 2). It remained in the reduced models. In the reduced models, long-term sick leave (>39 days) predicted a higher postoperative QuickDASH score.

About 1/3 of cases of the working age were unemployed during the year of surgery (Table 1). Unemployment did not affect postoperative QuickDASH scores (Table 2).

In the study population, 589/1346 (44%) had received social assistance at some point (Table 1). Of these, 114/589 (19%) had received it once, and 475/589 (81%) had received it more than once. Social assistance did not affect postoperative QuickDASH scores (Table 2).

## 4. Discussion

The main finding of this study was that the study population in general was socioeconomically deprived as defined by low education levels, low earnings, high dependence on social assistance, high unemployment rate, and high sick leave. All these factors might affect surgery outcome, but when adjusting for other socioeconomic factors, only long-term sick leave seemed to affect the patient-reported outcome.

Socioeconomic factors are important determinants in an individual's health and even mortality [13]. Only 25% of the population had more than 12 years of education. To put this in perspective, in 2016, 41% of the Swedish population between 25 and 64 years of age had more than 12 years of education [14]. In distal radius fractures, education is correlated both to postoperative DASH scores and postoperative range of motion, with higher education predicting both better subjective and objective outcomes [15].

In the present study, there was also a high proportion (44%) of the population that had received social assistance at least once during the years 1990-2016. Based on results from a study on the general Swedish population, approximately only 10% of the general population have received social assistance at least once [16]. In addition, about a third of the studied population in working age was unemployed at the time of surgery. As a comparison, 6-8% of the Swedish population were unemployed between the years 2010 and 2016. One other unexpected result in the present study is that being divorced was associated with a better outcome. This is contradictory to previous studies on other conditions. In cardiac surgery, being divorced, separated, or widowed was associated with 40% greater odds of death or new disability within the first two years after surgery [17]. It is possible that after surgery for ulnar nerve compression, the patient is not to the same extent dependent on spouses and relatives as after a major surgery. One shortcoming of our data is that we had no record of people living together with their partners whilst unmarried, a relationship form that is common in Sweden, which might obscure the results.

Two other factors, occupational factors and immigrant status, that one may consider influencing the occurrence of ulnar nerve compression and results of surgery did not show any signs of being important. Occupational factors, e.g., if the patient has a profession that includes manual labor, are often mentioned in studies as a risk factor for the development of ulnar nerve compression [5, 18, 19]. However, we did not find any evidence that manual labor contributed to worse outcome following surgery. Furthermore, immigrant status might also be a factor contributing to inequalities in surgical outcomes [20]. We did not however find any indication that so would be the case in ulnar nerve compression, in contrast to carpal tunnel syndrome [21].

Socioeconomic status is known to influence a number of other conditions [8]. In comparison to patients suffering and treated for another more common nerve compression lesion, carpal tunnel syndrome, our population was more socioeconomically deprived. This fact, in combination with the finding that outcomes were not very favorable using the QuickDASH, raises concerns about treatment indications. One study found, in a European population of approximately 25,000 individuals, that arm or hand pain was more prevalent in individuals with a low education level compared to individuals with a higher level of education [22]. Diagnosis of ulnar nerve compression is based on the patient history and the clinical examination, often with electrophysiology as a complement although its utility for diagnosis and predicting outcome may be debated [23].

In a US population, private insurance, which might indicate higher socioeconomic status, was associated with faster evaluation and surgical treatment for ulnar nerve compression than patients with public insurance [24]. In Sweden, the vast majority of health care is publicly funded, but it is possible that patients with low socioeconomic status and low health literacy do not seek, or seek care at a later stage, or have less access to health care, leading to a delayed diagnosis and treatment, possibly to a stage where surgical intervention is unavoidable or when severe and irreversible symptoms have developed. A higher preoperative McGowan score (used to classify sensorimotor deficit in ulnar nerve compression, i.e., higher score means greater deficit) is associated with worse postoperative DASH results [6]. Thus, part of the explanation as to why such a large proportion of this surgically treated population was socioeconomically deprived could be that their compression was severe at presentation.

In total, our population with surgically treated ulnar nerve compression seems to be different from the general population in several aspects. A high proportion of the population was unemployed, received social assistance, and had low education and low earnings. However, we did not find any conclusive evidence regarding the effect of socioeconomic factors on the outcome of surgery for ulnar nerve compression. The only variable that seemed to matter was long-term sick leave by all causes (i.e., not adjacent to the surgery). This might be obscured by the fact that many patients in our study had a low socioeconomic status but is in accordance with one previous study that found no association between socioeconomic status (based solely on occupation) and ulnar nerve compression [4]. It is also possible that comorbidities, such as shoulder or neck pain, are present to a higher degree in this population than in the general population, contributing to the high rate of sick leave and to the fact that long-term sick leave was associated with worse results. Outcomes after surgery, as measured by the QuickDASH, were not impressive in any of the groups, in agreement with our previously published data. When looking at absolute numbers, it seemed that education level and level of earned income might affect outcomes. This result, however, did not gain statistical significance in the multivariate regression model, suggesting that unfavorable outcomes have a multifaceted background. Thus, the patients' whole situation should be taken into consideration when planning appropriate treatment and rehabilitation.

The major strengths of this study are the large number of patients and the various socioeconomic factors included. The main limitation is the response rate, which was around 30%. This might introduce a selection bias, even though we did not find any large differences between responders and nonresponders.

## 5. Conclusion

Patients having surgery for ulnar nerve compression greatly differ from the general Swedish population concerning socioeconomic factors, but only a history of long-term sick leave influences the outcome of surgery. The patients' whole situation should be taken into consideration when planning appropriate treatment and rehabilitation.

## Figures and Tables

**Table 1:** Socioeconomic factors in surgically treated cases with ulnar nerve compression at the elbow divided into age categories.

	18-42 (*n* = 341)	43-52 (*n* = 337)	53-62 (*n* = 349)	>63 (*n* = 319)	*p* value	All (*n* = 1346)
Sex, female *n* (%)	197 (58)	155 (46)^∗^	159 (46)	138 (43)	0.001	649 (48)
Born outside Sweden *n* (%)	53 (16)	59 (18)	78 (22)	34 (11)^∗^	0.001	224 (17)
Highest education level *n* (%)	95 (28)	77 (23)	79 (23)	79 (25)	0.35	330 (25)
Sick days/employed year Median (IQR)	19 (6-54)	16 (4-42)	16 (5-41)	6 (0-24)^∗^	<0.0001	13 (3-38)
Days as unemployed/year Median (IQR)	29 (9-56)	16 (3-40)^∗^	6 (0-30)^∗^	0 (0-6)^∗^	<0.0001	7 (0-32)
Unemployed during year of surgery *n* (%)	88 (26)	94 (28)	116 (33)	258 (81)^∗^	<0.0001	556 (41)
Ever received social assistance *n* (%)	170 (50)	173 (51)	156 (45)	90 (28)^∗^	<0.0001	589 (44)
Mean annual earnings, 1000 SEK Median (IQR)	160 (0-284)	199 (97-287)^∗^	198 (45-280)	147 (35-271)	0.002	183 (39-281)
Preoperative QuickDASH Median (IQR)	50 (25-66)	51 (27-68)	55 (35-66)	50 (32-63)	0.54	50 (30-66)
3 months postoperative QuickDASH Median (IQR)	30 (14-59)	32 (11-52)	25 (11-55)	34 (14-56)	0.63	32 (12-55)
12 months postoperative QuickDASH Median (IQR)	41 (22-60)	28 (11-50)	39 (15-57)	34 (11-62)	0.22	36 (14-57)
Change in QuickDASH 0-12 months Median (IQR)	12 (1-26)	14 (0-25)	7 (-2-19)	11 (5-21)	0.64	11 (0-21)

Data presented as *n* (%) or median (interquartile range; IQR), as stated. ^∗^*p* < 0.05 compared to the column to the left. Kruskal-Wallis test with subsequent Bonferroni corrections for multiple tests.

**Table 2:** Linear regression model analyzing the effect of socioeconomic factors on postoperative QuickDASH scores at 12 months in cases surgically treated for ulnar nerve compression at the elbow.

	Model 1 Unadjusted B-coefficient (95% CI)	*p* value	Model 2 Adjusted B-coefficient (95% CI) (adjusted for age, sex, and diabetes)	*p* value	Model 3 Adjusted B-coefficient (95% CI) All variables included	*p* value
Age at surgery (years)	0.08 (-0.11-0.27)	0.41	0.12 (-0.08-0.31)	0.24	0.30 (-0.09-0.68)	0.13
Sex (male is reference)	8.96 (3.39-14.5)	0.002	9.6 (3.96-15.3)	0.001	8.96 (2.17-15.75)	0.01
Diabetes at surgery	1.30 (-7.16-9.77)	0.76	2.15 (-6.34-10.6)	0.62	0.60 (-8.43-9.64)	0.90
*Marital status*						
Not married (reference)						
Married	1.29 (-5.27-7.84)	0.70	-0.32 (-7.59-6.94)	0.93	-3.8 (-12.2-4.6)	0.37
Divorced	-2.46 (-10.7-5.81)	0.56	-5.45 (-14.5-3.61)	0.24	-12.6 (-22.6-2.6)	0.014
Widowed	12.6 (-1.51-26.7)	0.08	7.77 (-8.10-23.7)	0.34	1.42 (-14.7-17.6)	0.86
*Level of education*						
Low (reference)						
Middle	-4.80 (-12.0-2.42)	0.19	-4.0 (-11.3-3.3)	0.28	-0.91 (-9.1-7.3)	0.83
High	-12.5 (-20.6-4.4)	0.003	-12.3 (-20.4-4.2)	0.003	-7.62 (-16.8-1.57)	0.10
*Earnings (mean/year)*						
≤39,100 (reference)						
39,200-183,100	-3.29 (-11.17-4.60)	0.41	-4.13 (-12.0-3.73)	0.30	-7.1 (-19.7-5.52)	0.27
183,200-280,700	-10.2 (-18.2-2.22)	0.12	-10.5 (-18.5-2.54)	0.01	-6.16 (-19.0-6.70)	0.35
>280,800	-15.0 (-23.0-7.0)	<0.0001	-13.8 (-21.8-5.76)	0.001	-8.45 (-21.1-4.19)	0.19
*Migrant status*						
Born in Sweden (reference)						
Born outside of Sweden	5.08 (-2.72-12.89)	0.20	5.96 (-1.76-13.7)	0.13	0.16 (-10.2-10.6)	0.98
*Occupation*						
Nonmanual (reference)					-3.13 (-10.1-3.84)	0.38
Manual	-2.21 (-8.19-3.77)	0.47	-1.51 (-7.47-4.44)	0.62	-3.13 (-10.1-12.3)	0.38
*Sick leave*						
0 days (reference)						
1-13 days	-14.0 (-23.45-4.58)	0.004	-11.2 (-20.8-1.61)	0.02	-7.2 (-18.0-3.79)	0.20
14-38 days	-3.84 (-13.76-6.08)	0.45	-1.80 (-11.9-8.3)	0.73	0.68 (-10.9-12.3)	0.91
>39 days	4.27 (-6.18-14.72)	0.42	7.82 (-3.13-18.8)	0.16	11.9 (-0.88-24.7)	0.068
*Unemployment*						
Mean days/year	-0.14 (-0.14-0.11)	0.82	0.078 (-0.057-0.213)	0.26	-0.16 (-0.17-0.14)	0.83
*Social assistance*						
Never received (reference)						
Received once	3.46 (-6.07-12.99)	0.48	4.02 (-5.5-13.6)	0.41	3.66 (-7.40-14.71)	0.52
Received more than once	5.53 (-0.91-12.0)	0.09	6.05 (-0.53-12.6)	0.07	3.15 (-5.64-11.93)	0.48

**Table 3:** Reduced model 1. Reduced linear regression model analyzing the effect of socioeconomic factors on postoperative QuickDASH scores at 12 months in cases surgically treated for ulnar nerve compression at the elbow. Variables with a *p* < 0.3 in the original model are included.

	Unadjusted B-coefficient (95% CI)	*p* value
Age at surgery (years)	0.32 (0.06-0.59)	0.018
Sex (male is reference)	8.88 (2.65-15.0)	0.005
*Marital status*		
Not married/married/widowed (reference)		
Divorced	-9.8 (-17.5-2.18)	0.012
*Level of education*		
Low/middle (reference)		
High	-6.58 (-13.6-0.48)	0.068
*Earnings (mean/year)*		
≤39,100/183,200-280,700 (reference)		
39,200-183,100	-3.27 (-10.5-3.92)	0.37
>280,800	-4.9 (-12.5-2.65)	0.20
*Sick leave*		
0 days/14-38 days (reference)		
1-13 days	-9.77 (-16.5-3.0)	0.005
>39 days	8.48 (0.055-16.9)	0.049

Excluded variables: diabetes at surgery, migrant status, occupation, unemployment, and social assistance.

**Table 4:** Reduced model 2. Reduced linear regression model analyzing the effect of socioeconomic factors on postoperative QuickDASH scores at 12 months in cases surgically treated for ulnar nerve compression at the elbow. All variables with *p* value < 0.1 in reduced model 1 are included.

	Unadjusted B-coefficient (95% CI)	*p* value
Age at surgery (years)	0.33 (0.069-0.60)	0.014
Sex (male is reference)	9.68 (3.70-15.7)	0.002
*Marital status*		
Not married/married/widowed (reference)		
Divorced	-10.2 (-17.8-2.57)	0.009
*Level of education*		
Low/middle (reference)		
High	-6.57 (-13.5-0.38)	0.064
*Sick leave*		
0 days/14-38 days (reference)		
1-13 days	-9.9 (-16.6-3.2)	0.004
>39 days	9.12 (0.89-17.3)	0.03

Excluded variables: diabetes at surgery, migrant status, occupation, unemployment, social assistance, and earnings.

## Data Availability

Public access to the data is restricted by the Swedish Authorities (Public Access to Information and Secrecy Act; http://www.government.se/information-material/2009/09/public-access-to-information-and-secrecy-act/), but data can be made available for researchers after a special review that includes approval of the research project by both an Ethics Committee and the authorities' data safety committees.

## References

[1] Caliandro P., La Torre G., Padua R., Giannini F., Padua L. (2016). Treatment for ulnar neuropathy at the elbow. *Cochrane Database of Systematic Reviews*.

[2] Mondelli M., Giannini F., Ballerini M., Ginanneschi F., Martorelli E. (2005). Incidence of ulnar neuropathy at the elbow in the province of Siena (Italy). *Journal of the Neurological Sciences*.

[3] Osei D. A., Groves A. P., Bommarito K., Ray W. Z. (2017). Cubital tunnel syndrome: incidence and demographics in a national administrative database. *Neurosurgery*.

[4] Hulkkonen S., Auvinen J., Miettunen J., Karppinen J., Ryhanen J. (2019). Smoking is associated with ulnar nerve entrapment: a birth cohort study. *Scientific Reports*.

[5] Bartels R. H., Verbeek A. L. M. (2007). Risk factors for ulnar nerve compression at the elbow: a case control study. *Acta Neurochirurgica*.

[6] Giöstad A., Nyman E. (2019). Patient characteristics in ulnar nerve compression at the elbow at a tertiary referral hospital and predictive factors for outcomes of simple decompression versus subcutaneous transposition of the ulnar nerve. *BioMed Research International*.

[7] Anker I., Andersson G., Zimmerman M., Jacobsson H., Dahlin L. B. (2019). Subcutaneous and submuscular transposition due to ulnar nerve entrapment at the elbow–analyses of 43 primary and 44 revision cases. *Hand Microsurg.*.

[8] Li X., Galvin J. W., Li C., Agrawal R., Curry E. J. (2020). The impact of socioeconomic status on outcomes in orthopaedic surgery. *The Journal of Bone and Joint Surgery. American Volume*.

[9] The Swedish translated version of Quick DASH. http://www.dash.iwh.on.ca/assets/images/pdfs/QuickDASH_Swedish.pdf.

[10] United Nations Educational Scientific and Cultural Organization (UNESCO) Institute for Statistics. ISCED mappings 2011. http://unesco.org/en/isced-mappings.

[11] Rosberg H. E., Carlsson K. S., Hojgard S., Lindgren B., Lundborg G., Dahlin L. B. (2016). What determines the costs of repair and rehabilitation of flexor tendon injuries in zone II? A multiple regression analysis of data from southern Sweden. *Journal of Hand Surgery*.

[12] Zimmerman M., Anker I., Karlsson A. (2020). ulnar nerve entrapment in Diabetes: Patient-reported outcome after Surgery in national quality registries. *Plast Reconstr Surg Glob Open*.

[13] Lantz P. M., House JS, Lepkowski J. M., Williams D. R., Mero R. P., Chen J. (1998). Socioeconomic factors, health behaviors, and mortality: results from a nationally representative prospective study of US adults. *Journal of the American Medical Association*.

[14] Statistics Sweden http://www.scb.se/hitta-statistik/statistik-efter-amne/utbildning-och-forskning/befolkningens-utbildning/befolkningens-utbildning/.

[15] Paksima N., Pahk B., Romo S., Egol K. A. (2013). The association of education level on outcome after distal radius fracture. *The Hand*.

[16] Mood C. (2013). Social assistance dynamics in Sweden: duration dependence and heterogeneity. *Social Science ResearchSocial Science Research*.

[17] Neuman M. D., Werner R. M. (2016). Marital status and postoperative functional recovery. *JAMA Surgery*.

[18] Descatha A., Leclerc A., Chastang J. F., Roquelaure Y. (2004). Incidence of ulnar nerve entrapment at the elbow in repetitive work. *Scandinavian Journal of Work, Environment & Health*.

[19] Fadel M., Lancigu R., Raimbeau G., Roquelaure Y., Descatha A. (2017). Facteurs pronostiques professionnels dans la compression du nerf ulnaire au coude : revue systematique. *Hand Surgery and Rehabilitation*.

[20] Krupic F., Garellick G., Gordon M., Karrholm J. (2014). Different patient-reported outcomes in immigrants and patients born in Sweden: 18, 791 patients with 1 year follow-up in the Swedish Hip Arthroplasty Registry. *Acta Orthopaedica*.

[21] Zimmerman M., Hall E., Steen Carlsson K., Nyman E., Dahlin L. B. (2019). *Socioeconomic factors predicting outcome after open carpal tunnel release – a national registry-based study*.

[22] McNamara C. L., Balaj M., Thomson K. H., Eikemo T. A., Solheim E. F., Bambra C. (2017). The socioeconomic distribution of non-communicable diseases in Europe: findings from the European Social Survey (2014) special module on the social determinants of health. *European journal of public health*.

[23] Shi Q., Mac Dermid J. C., Santaguida P. L., Kyu H. H. (2011). Predictors of surgical outcomes following anterior transposition of ulnar nerve for cubital tunnel syndrome: a systematic review. *The Journal of Hand Surgery*.

[24] Cheng C., Rodner C. M. (2020). Associations between insurance type and the presentation of cubital tunnel syndrome. *The Journal of Hand Surgery*.

